# A case report and literature review on nephrogenic alveolar soft part sarcoma: clinicopathological manifestations and genetic features

**DOI:** 10.1186/s12894-023-01182-0

**Published:** 2023-02-13

**Authors:** Xue Wang, Jingjing Yu, Xiaodong Fan, Suya Ma, Xiaohong Xie, Ning Gao, Shuaishuai Huang, Aimei Lu

**Affiliations:** 1 Department of Ultrasound, Ningbo Yinzhou Second Hospital, Ningbo, 315000 Zhejiang People’s Republic of China; 2 Department of Pathology, Ningbo Yinzhou Second Hospital, Ningbo, 315000 Zhejiang People’s Republic of China; 3 Department of Urologic Surgery, Ningbo Yinzhou Second Hospital, Ningbo, 315000 Zhejiang People’s Republic of China; 4 Tumor Companion Diagnosis Broad Market Business Unit, 3D Medicines Inc., Shanghai, People’s Republic of China

**Keywords:** Nephrogenic, Alveolar soft part sarcoma, Clinical manifestation, Genetic, TFE3

## Abstract

**Background:**

Alveolar soft part sarcoma (ASPS) is a rare kind of malignant soft tissue tumor with undefined differentiation, of which the incidence rate accounts for only 0.5–1.0% among all kinds of soft tissue tumors. An even rarer ASPS occurs in kidney.

**Case presentation:**

Here we reported a case of a 7-year-old girl diagnosed with nephrogenic ASPS, regarding the analyses of the incidence, clinical manifestation, pathology and genetic diagnosis, in order to deepen the recognition of the disease.

**Conclusions:**

ASPS is very rare, and tends to occur to young patients. It is very significant to precisely diagnose ASPS at an early stage, which will be the key point for the following treatment choices and prognosis.

## Background

Alveolar Soft Part Sarcoma (ASPS), as a malignant tumor with unclear differentiation, tends to occur in juveniles, with the age of peak onset ranging from 15 to 35 and the histology featuring as specific acinus-like or organ-like structures [1]. ASPS usually appears as a kind of soft tissue mass with painless slow growth, which is easy to be neglected. Among the literature, nephrogenic ASPS was only reported in one publication by Kim et al. in [2]. This study presents the second case of such a rare entity, and reviews the relevant literature, and focuses on the differential diagnosis from other renal tumors, so as to cause suspicions from clinicians and pathologists.

## Case presentation

A 7-year-old female patient came to see a doctor in our hospital for low back ache with gross hematuria for one day. No abnormality was detected by physical examination. The result of routine urinalysis indicated 2083 RBCs/µl, while no abnormality was reported in other laboratory examinations.

**Fig. 1:**
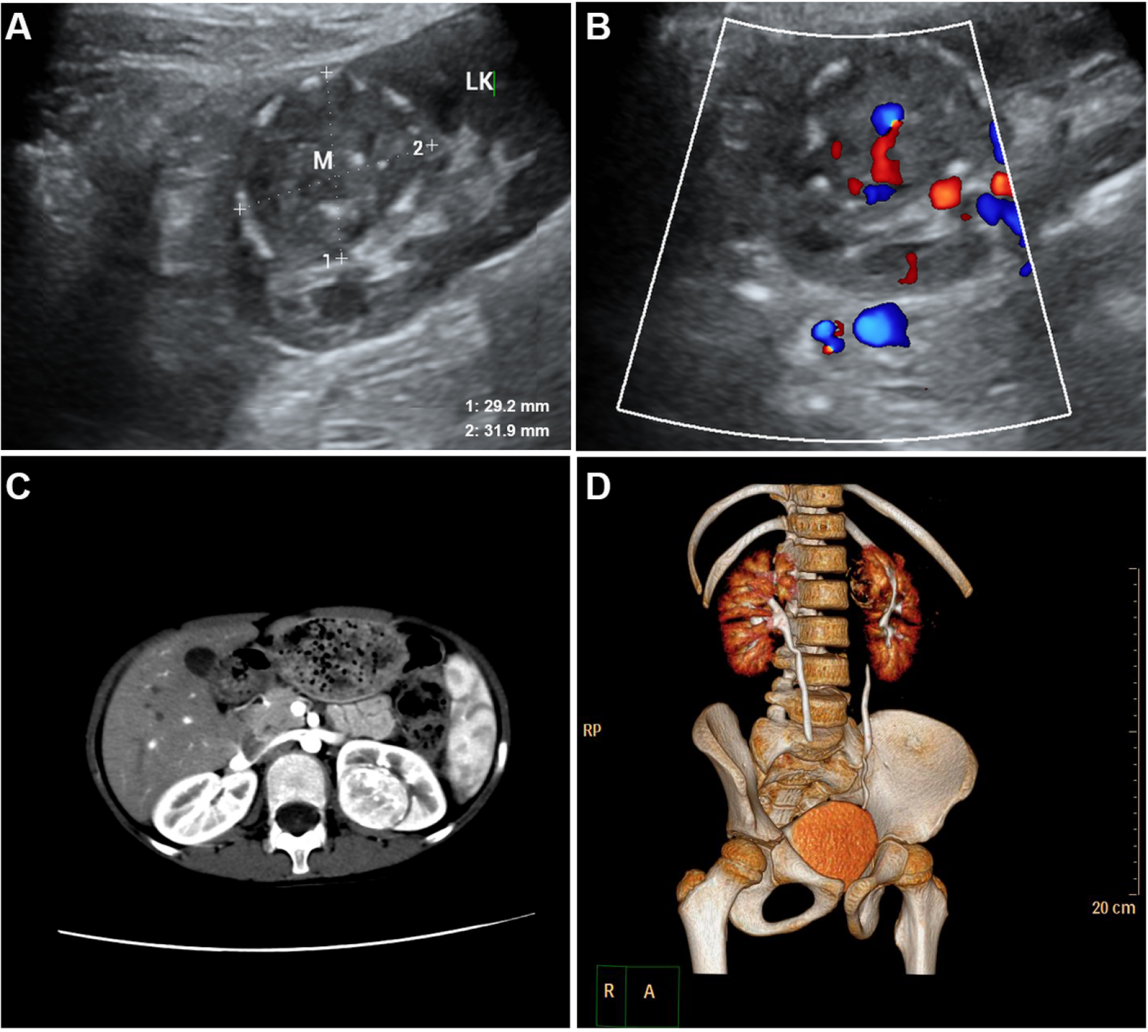
Two-dimensional ultrasound showed a solid hypoechoic mass at the upper level of the left kidney with clear boundary and annular calcification around (**A**). CDFI showed abundant blood flow signals (**B**). Contrast enhanced CT showed heterogeneous enhancement mass of left renal parenchyma (**C**). 3D imaging showed that the mass was located on the superior dorsal side of the left kidney, and some renal calyces were compressed and narrowed (**D**)

Ultrasonic examination (Mindray Resona 7) indicated: A solid hypoechoic mass with a size of 29.2 × 31.9 mm located in the dorsal parenchyma of upper pole of left kidney, with clear border and being endogenous for most part, slightly protruding from the surface of kidney, partly pressing towards renal collecting system, and annular calcification was observed in its surrounding (Fig. 1A). Results from CDFI showed abundant and coarse blood signals inside of the mass (Fig. 1B).

CTU inspection (GE Light Speed 64 CT) showed a circular soft tissue density with a size of 29 × 32 mm, which exhibited non-uniform interior and multiple arc and spot-like shadows of calcification inside of the foci and in the surrounding. The CT value of the solid part was 44HU, which was obviously increased after enhanced scanning. CT value during renal cortex phase was approximately 155 HU which equaled to that during renal medullary phase. Coarse shadows of blood vessels were observed inside of the foci, with a few small spot-like non-intensified focus in the center. The border between the mass and renal tissue was clear, and no apparent symptom of destruction was observed in neighbouring kidney calices (Fig. 1C). Three-dimensional (3D) imaging displayed that bilateral renal pelvis and calices were well imaged, in which left renal calices was narrowed by pressure. Bilateral ureters were unobstructed with no obvious filling-defect observed (Fig. 1D). Image diagnosis of the case was ‘left renal mass with the consideration of malignant tumor, nephroblastoma?” Radical nephrectomy of the left kidney was suggested after the completion of other related inspections.

**Fig. 2:**
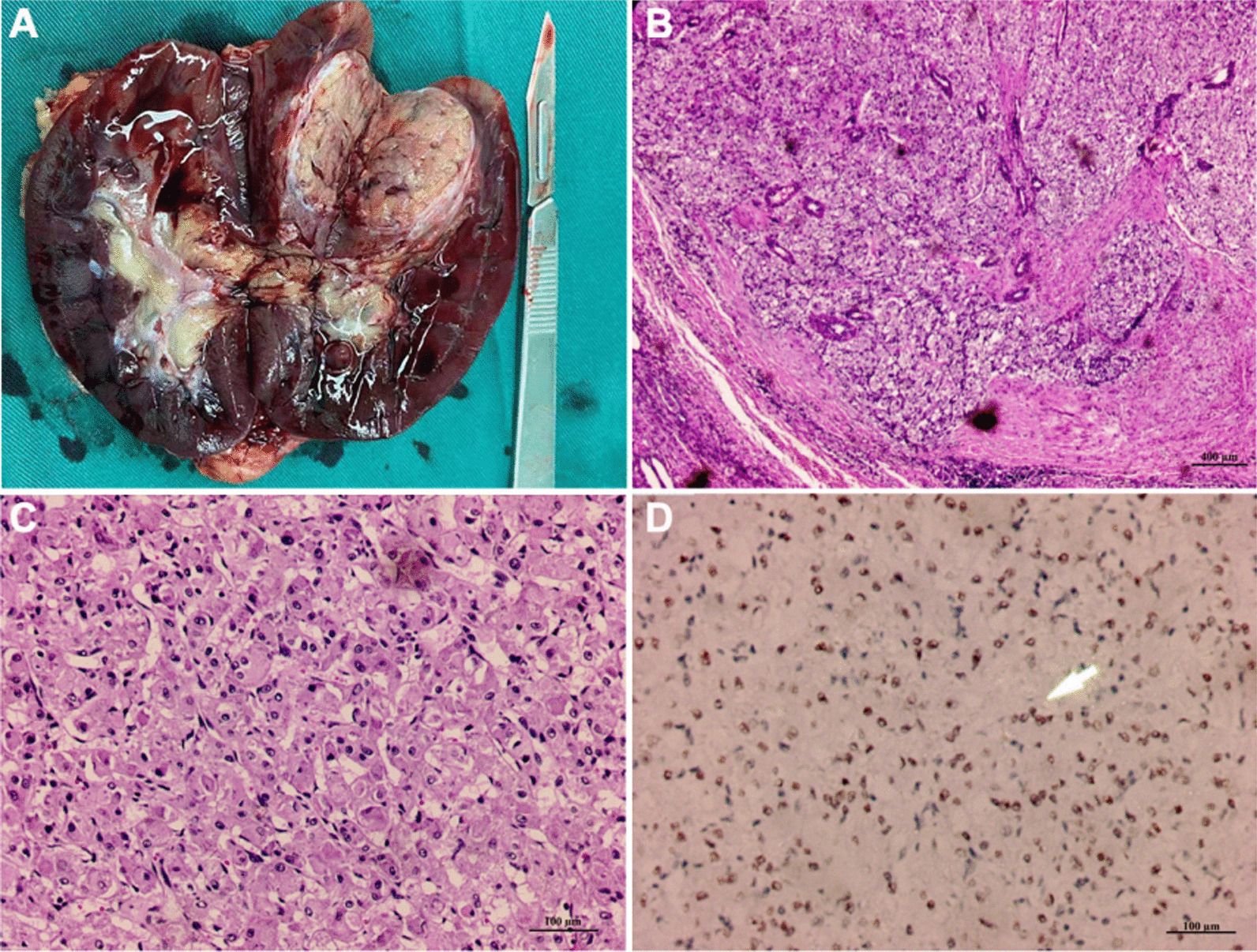
After operation, a grayish yellow tumor with capsule was shown at the upper pole of the left kidney (**A**). At low magnification, tumor cells are seen in nodular arrangement (**B**). The cell volume is large, the cytoplasm is rich, eosinophilic (**C**). Immunohistochemistry showed that the nucleus was TFE3 positive (**D**). Pathological section was scanned by Magscanner (KFBIO, Ningbo, China)

Observations during the surgery: Radical nephrectomy of the left kidney was performed. Exposing the mass by cutting though the kidney, we observed the integrate capsule wrapping round the surface of the mass. When the capsule was cut open, the section of the mass was solid, white and medium-hard, with slight necrosis and hemorrhage inside. Gross observation: a grayish yellow mass located on the upper pole of the section of left kidney, clinging to the renal cortex, with clear border, the capsule, and a size of 29 mm × 32 mm × 22 mm (Fig. 2A).

Postoperative pathological results: observed under microscope at low magnification, tumor cells were arranged in a solid or nodular pattern, and the interfibrillar substances between cell nests were rich in blood vessels, as shown in Fig. 2B (40×); at high magnification, tumor cells were large in size and rich in acidophily cytoplasm, and had prominent nucleus with scarce nuclear fission, as shown in Fig. 2C (200×). Immunohistochemistry results: TFE3 nucleus (+) (Fig. 2D), SDH-B (−), VHL (−), CAIX (−), CK7 (−), CD117 (−), CD34 (sinusoids+), MyoD1(−), Myogenin (−), Myoglobin (−), Desmin (−), PAX8 (−), CK pan (−), NSE(+), SOX-10 (−), CgA (−), Syn (−), S-100 (−), HMB45 (−), SMA (sinusoids+), PNL2 (−), CathepsinK (+), SF-1 (−), BCOR (−), Vimentin (+), Melan A (−), Ki-67 (+) l%. In combination with cell morphology and immunohistochemistry results, it was deduced to be ASPS. After systemic examination, no obvious lesions were found in other parts except the left kidney, which is considered to be nephrogenic ASPS.

**Fig. 3:**
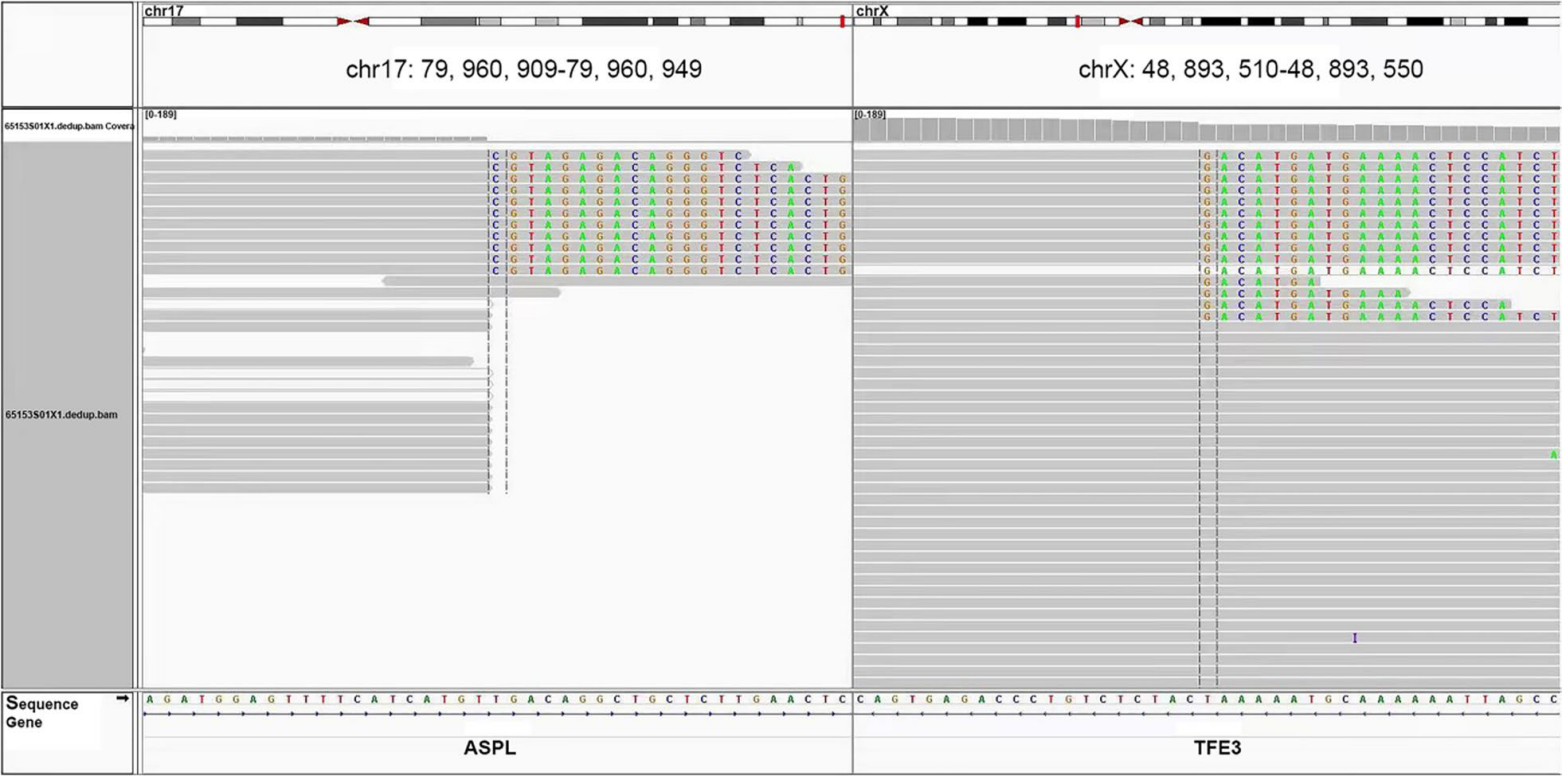
Gene sequencing showed that ASPSCR1 gene on chromosome 17 was rearranged with TFE3 gene on chromosome X to form ASPSCR1-TFE3 fusion gene

Detection of genetic variation: Using fluorescence in situ hybridization method (FISH), ectopia was detected in t(Xp11.2) (TFE-3) genes. Gene sequencing was performed at 3D Med Inc. The results shown that gene arrangement occurred between exons 1–7 of ASPL and exons 6–10 of TFE3 (Fig. 3). Gene fusion of ASPL-TFE3 further verified the accuracy of this case as ASPS.

## Discussion and conclusions

In 1952, 12 cases of malignant soft tissue sarcoma were reported by christopherson et al.3 and termed as ASPS. Being a kind of rare soft tissue sarcoma, ASPS tends to occur in head-and-neck region of children and adult’s limbs and trunk [4]. A total of 74 cases of ASPS reported in 42 studies published from 1957 to 2018 were retrospectively analyzed by Skele et.al, in which male patients accounted for 41.9% (31 cases), while females accounted for 58.1% (43 cases), and the average age of them were 18.25 ± 15.243; there were 60 cases of ASPS occurred in oral cavity, including 42 cases in tongue (70%), 9 cases in cheek (15%), 4 cases in lower jawbone (6.66%), 2 cases in gingiva (3.33%), one case in lips (1.66%), one in buccal space (1.66%) and one in buccal mucous membrane (1.66%) [5]. Despite the multi-locations of ASPS’ occurrence, there was, by far, only one reported case which occurred in kidney all over the world [2]. In clinic, ASPS usually appears as a kind of slow-growing painless mass, which is easily neglected by patients and doctors. In this case, the tumor which was originally thought to be nephroblastoma was not diagnosed to be ASPS until pathological tests were conducted after the mass was removed by radical nephrectomy.

The ultrasonic feature of ASPS is usually hypoechoic node with clear border and abundant blood signal inside. CT imaging shows soft tissue mass with density shadows similar/lower than muscle tissue, and the contrast can be obviously enhanced by iodic contrast agent [6]. MRI indicates equal or slightly higher signals in T1W1 and uneven high signals in T2W1 [7]. The patient in this case presented the same features in ultrasound imaging and CT imaging, but did not accept MRI inspection.

Given the specific morphological features, the golden criterion for the diagnosis of ASPS is pathological diagnosis. In morphology, ASPS tumor cells are usually arranged in acinus-like or tubular pattern, and cell nests are separated by fibrous connective tissues, were fissuring-shaped or sinus-shaped capillary network with rich flat endothelium can be observed. The tumor cells are big sphere or polygon, with clear border, vacuolus nucleus, prominent nucleoli and acidophilic cytoplasm. Tumor cells were PSA-positive, in which aubergine needle/rod-like crystal substance can be observed, and they are resistant to amylase digestion; Flores et.al have found that ASPS tumor cells in infants and children may grow in solid/diffusible patterns without nest-like structures. In this case, we found that the growth of this nephrogenic ASPS happened to be in solid pattern in which tumor cells arranged in nodositas without obvious acinous structure, however, the segregation between blood-sinus-like capillary network and fibrous septum was apparent.

It has been a controversy about the histologic origin of ASPS tumor cells, and the differential diagnosis with other tumors always needs evidences based on multiple immunohistochemistry markers. For most malignant tumor cells, Ki-67 accounts for a high positive expression rate, whereas ASPS presents relatively low. Consistently, Ki-67 was at low expression in this case.

Furthermore, granular cell tumor expresses S-100 and SOX10 [8]; malignant melanoma expresses HMB45, Melan-A and S-100 [9]; alveolar rhabdomyosarcoma expresses Desmin and MyoD1 [10]; paraganglioma expresses Syn, S-100 and SOX10 [11]; PEComa expresses HMB-45, Melan-A, SMA, Desmin and Calponin [12]; clear cell sarcoma of soft tissue expresses S-100, SOX10, HMB-45 and Melan-A [13]; metastatic ccRCC expresses CK(p), EMA and CD10 [14]. Regarding this case, S-100, SOX10, Melan-A, S-100, Desmin, MyoD1, Syn, HMB45, Melan-A, CK (p) and CD10 were all negative while only Ki-67 and SMA was positive, based on which these common soft tissue tumors mentioned above were excluded. In addition, TFE3 is recognized as a specific marker for the diagnosis of ASPS [6]. In this case, TFE3 (located in the nucleus) was positive. Taken this and results of tests on other immunochemistry markers, this case was finally diagnosed as ASPS.

Lagodziska et. al have reported that t(X; 17)(p11.2; q25) chromosome translocation occurred in ASPS, which meant that a new fusion gene *ASPL-TEF3* was formed by an unbalanced translocation between TEF3 transcription factor gene on chromosome site *X*p11.2 and *ASPL* gene on chromosome site 17q25, inducing abnormal proliferation of cells thus tumorigenesis [15]. Notably, we also detected the formation of new fusion gene *ASPL-TEF3* in our case, in which genetic rearrangement occurred to exon 1–7 of ASPL and exon 6–10 of TFE3 (Fig. 3). The mRNA of *ASPL-TEF3* gene can also be detected in some renal cell carcinomas, however, distinguished from the balanced translocation in renal cell carcinoma, the ASPS was unbalanced [16].

Otherwise, mutations of *KMT2C* and *BRCA2* were found, whereas the gene of *ALK, BRAF, CD274, EGFR, FGFR2* and *FGFR3* were normal. In *BRCA2*-mutated cells, the repair ability of DNA double-strand damage was impaired, and the express deficiency of BRCA is related to the occurrence and progression of non-small cell lung cancer, pituitary tumor, thyroid cancer, pancreatic cancer, as well as breast cancer [17, 18]. However, no literature regarding on whether BRCA2 mutations are associated with ASPS has ever been published, therefore more case analyses are required. KMT2C(MLL3), a tumor suppressor gene, is intimately associated with lung cancer, colon cancer, breast cancer and bladder cancer [19]. In this case, mutation was detected in c.7443-1delinsAT of KMT2C, where a change in the standard receptor of splicing site occurred to intron 37th of KMT2C, causing the expression deficiency of KMT2C [20]. Previous study has demonstrated that RAS signaling pathway might be highly activated and p53 might be inactivated due to the lack of KMT2C [21]. Nevertheless, it is still unclear regarding the relationship between the loss of KMT2C and ASPS.

Compared with other malignant tumors, ASPS possesses worse prognosis and higher invasion. Factors that affect its prognosis include age of onset, tumor size, primary site and metastasis. It has been considered by most researchers that the key point of treating ASPS is radical surgery. Given its poor prognosis and unremarkable effects of adjuvant chemoradiotherapy, molecular targeted therapy has become a new choice [22]. However, more clinical evidences are required to prove the safety and effectiveness of these therapies. Here, we consider the young age of this patient and early diagnosis of ASPS and metastasis-not-found yet, molecular targeted therapy has not been performed. With the follow-up lasting for 18 months by now, no recurrence or metastasis was detected, the follow-up will be continued.

To sum up, ASPS is very rare, and tends to occur to young patients. It is very significant to precisely diagnose ASPS at an early stage, which will be the key point for the following treatment choices and prognosis.

## Data Availability

All data related to this study are publicly available at https://ngdc.cncb.ac.cn/gsa-human/browse/HRA003345.

## Abbreviations

ASPS: Alveolar soft part sarcoma
3D: Three-dimensional
FISH: Fluorescence in situ hybridization method
